# Tribbles 2 pseudokinase confers enzalutamide resistance in prostate cancer by promoting lineage plasticity

**DOI:** 10.1016/j.jbc.2021.101556

**Published:** 2021-12-30

**Authors:** Jitender Monga, Indra Adrianto, Craig Rogers, Shirish Gadgeel, Dhananjay Chitale, Joshi J. Alumkal, Himisha Beltran, Amina Zoubeidi, Jagadananda Ghosh

**Affiliations:** 1 Vattikuti Urology Institute, Henry Ford Health System, Detroit, Michigan, USA; 2 Public Health Sciences, Henry Ford Health System, Detroit, Michigan, USA; 3 Henry Ford Cancer Institute, Henry Ford Health System, Detroit, Michigan, USA; 4 Department of Pathology, Henry Ford Health System, Detroit, Michigan, USA; 5 Department of Internal Medicine, Univeristy of Michigan Rogel Cancer Center, Ann Arbor, Michigan, USA; 6 Department of Medical Oncology, Dana Farber Cancer Institute and Harvard Medical School, Boston, Massachusetts, USA; 7 Department of Urologic Sciences, University of British Columbia and The Vancouver Prostate Centre, Vancouver, British Columbia, Canada

**Keywords:** TRIB2, pseudokinase, enzalutamide resistance, prostate cancer, lineage plasticity, neuroendocrine, transdifferentiation, AFA, Afatinib, AR, androgen receptor, BRN2, Brain-2, CHGA, Chromogranin-A, CK-8, cytokeratin 8, NE, neuroendocrine, NSE, Enolase-2, SOX2, SRY-box 2, SYP, Synaptophysin, TMA, tissue microarray, TRIB2, Tribbles 2, TRIB2-OE, TRIB2-overexpressing

## Abstract

Enzalutamide, a second-generation antiandrogen, is commonly prescribed for the therapy of advanced prostate cancer, but enzalutamide-resistant, lethal, or incurable disease invariably develops. To understand the molecular mechanism(s) behind enzalutamide resistance, here, we comprehensively analyzed a range of prostate tumors and clinically relevant models by gene expression array, immunohistochemistry, and Western blot, which revealed that enzalutamide-resistant prostate cancer cells and tumors overexpress the pseudokinase, Tribbles 2 (TRIB2). Inhibition of TRIB2 decreases the viability of enzalutamide-resistant prostate cancer cells, suggesting a critical role of TRIB2 in these cells. Moreover, the overexpression of TRIB2 confers resistance in prostate cancer cells to clinically relevant doses of enzalutamide, and this resistance is lost upon inhibition of TRIB2. Interestingly, we found that TRIB2 downregulates the luminal markers androgen receptor and cytokeratin 8 in prostate cancer cells but upregulates the neuronal transcription factor BRN2 (Brain-2) and the stemness factor SOX2 (SRY-box 2) to induce neuroendocrine characteristics. Finally, we show that inhibition of either TRIB2 or its downstream targets, BRN2 or SOX2, resensitizes resistant prostate cancer cells to enzalutamide. Thus, TRIB2 emerges as a potential new regulator of transdifferentiation that confers enzalutamide resistance in prostate cancer cells via a mechanism involving increased cellular plasticity and lineage switching.

Enzalutamide, an inhibitor of androgen receptor (AR) function, is a popular drug commonly prescribed to treat advanced prostate cancer, but resistant prostate cancer eventually develops which grow aggressively, leading to widespread metastatic disease and ends up with a lethal outcome (1, 2, 3). Based on present assessment, the enzalutamide resistant type of aggressive prostate cancer is responsible for most of the morbidity and mortality associated with prostate cancer and ∼30,000 lives of American men are lost every year (4). Nevertheless, the lack of proper understanding about critical molecular targets in hormone refractory, enzalutamide-resistant prostate cancer cells, largely contributes to majority of the prostate cancer fatalities. Clinical manifestation of drug resistance is the result of selective growth of cell clones that are either intrinsically capable of or have acquired the power to resist drug’s effects on critical survival mechanisms. However, knowledge about signaling mechanisms that play active roles in the progression phase of prostate cancer to enzalutamide resistance is still limited, which is a delaying progress toward the development of a long-term, effective therapeutic strategy.

Common forms of prostate cancer cells bear luminal characteristics and depend on androgenic signaling for survival and growth, which is the basis for antiandrogenic therapy. However, it has been realized that men with prostate cancer who were treated with antiandrogenic therapies frequently develop aggressive and deadly forms of prostate cancers, which are no longer responsive to androgen-blockade therapies. Several reports encompassing the involvement of both AR reactivation or bypass, as well as androgen-independent signaling, have been forwarded to explain the mechanism of enzalutamide resistance. However, analysis of multiple cell lines and *in vivo* models, which were used to explore the molecular basis, have ended up with identification of cancer cell subtypes (5, 6). Current molecular understanding suggests that in addition to AR reactivation by mutation or splice variants, manifestation of enzalutamide resistance can be the result of overgrowth of cells that are developed in the tumor by lineage switching which may be triggered by drug-induced repression or loss of the AR-signaling (7, 8, 9). About 10 to 20% of enzalutamide-resistant prostate cancers show neuroendocrine (NE) features and no effective treatments are currently available for this type of aggressive and highly invasive prostate cancer (10, 11, 12). Though the continued growth and metastasis of the heavily enzalutamide-treated prostate tumors can be driven by nonandrogenic signaling, molecular underpinnings of critical targetable mechanisms in treatment-emergent NE type prostate cancer cells are yet to be fully characterized.

When prostate cancer cells become resistant to strong androgen-receptor blockers, such as enzalutamide, their common characteristics change from slow-growing and noninvasive to fast-growing, highly invasive type, but the knowledge about critical signaling mechanisms driving rapid growth and resistance to enzalutamide is still limited. To better understand the mechanistic basis behind enzalutamide resistance, we developed an *in vitro* model by chronically treating human LNCaP, MDA-PCa-2B, and LAPC4 prostate cancer cells (AR-signaling intact) with gradually increasing doses of enzalutamide (up to 30 μM) for >12 weeks to mimic the clinical conditions in standard long-term enzalutamide therapy (13). The resultant cells (LNCaP-ENR, PCa-2B-ENR, and LAPC4-ENR) are completely resistant to clinically relevant doses of enzalutamide, the blood level of which goes up to ∼34 μM in average (14, 15). By gene expression array, RT-PCR, and Western blot, we found that enzalutamide-resistant prostate cancer cells overexpress Tribbles 2 (TRIB2), a member of the Tribbles pseudokinase family (TRIB1-3). We also found that overexpression of TRIB2 alone, by gene transfection, can confer resistance to physiological doses of enzalutamide. Interestingly, molecular characterization revealed that the overexpression of TRIB2 suppresses luminal characteristics and induces NE features involving the master neuronal transcription factor, BRN2 (Brain-2), and the stemness regulator, SOX2 (SRY-box 2). Moreover, inhibition of either TRIB2 or its targets (BRN2 or SOX2) resensitizes resistant cells to enzalutamide. These findings indicate that TRIB2 is a new biomarker for NE-type prostate cancer and suggest that TRIB2 may contribute to enzalutamide resistance in prostate cancer cells, at least in part, by promoting lineage plasticity and phenotype switching.

## Results

### Enzalutamide-resistant prostate cancer cells and tumors overexpress TRIB2

**Figure 1:**
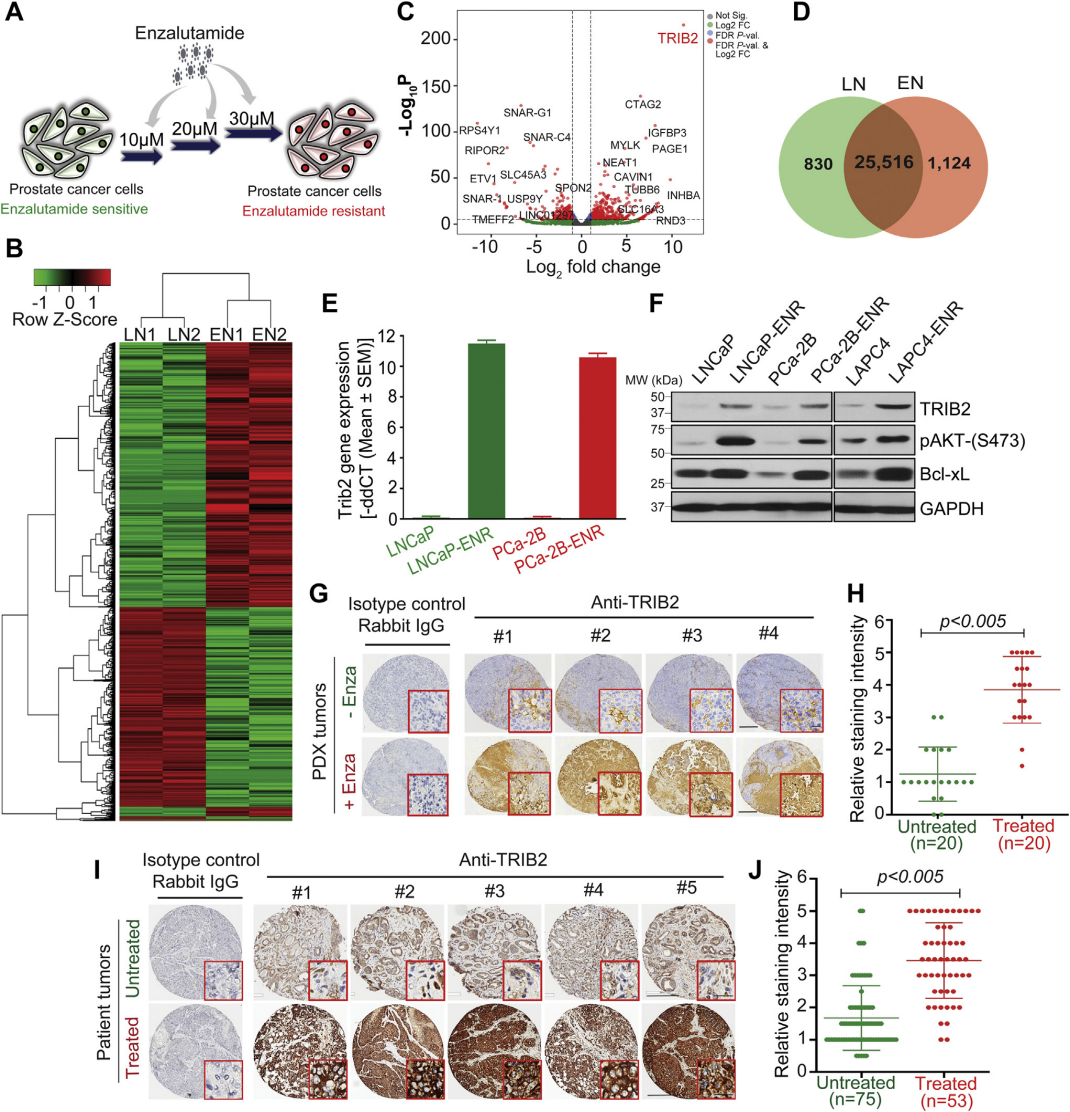
**Overexpression of TRIB2 in enzalutamide-resistant prostate cancer cells and tumors.***A*, a model depicting the strategy to develop enzalutamide-resistant prostate cancer cells *in vitro*. *B*, heatmap showing upregualted and downregulated genes in LNCaP-ENR cells (EN1 and EN2) compared to parental LNCaP cells (LN1 and LN2). *C*, volcano plot to show fold change in the levels of gene expression. *D*, Venn diagram showing unique and common expression of genes in parental and resistant cells. *E*, upregulation of TRIB2 mRNA in LNCaP-ENR and PCa-2B-ENR cells compared to parental cells by RT-PCR. *F*, Western blot showing increased protein levels of TRIB2 and targets in enzalutamide-resistant cells, compared to parental cells. Positions of the molecular weight markers for the ladder are indicated along with the Western blot data. *G* and *I*, representative IHC pictures (showing average expression) of TRIB2 staining intensity in prostate PDX (n = 20) and patient prostate tumor samples with (n = 53) or without (n = 75) enzalutamide treatment (*G*, the scale bar represents 100 μm; the scale bar represents 50 μm (*inset*); *I*, the scale bar represents 200 μm; the scale bar represents 25 μm (*inset*)). *H* and *J*, dot plot showing quantitation of TRIB2 expression level in prostate tumor samples using QuPath digital image analysis software. The statistical significance of the difference between untreated and treated samples (*H* and *J*) was calculated by two-tailed unpaired Student’s *t* test. IHC, immunohistochemistry; TRIB2, Tribbles 2.

A comprehensive gene expression array analysis revealed that the TRIB2 pseudokinase is grossly overexpressed in enzalutamide-resistant (EN1 and EN2) prostate cancer cells, compared to parental enzalutamide sensitive (LN1 and LN2) cells (Fig. 1, *A*–*D*). To confirm the array data, we detected TRIB2 mRNA expression by RT-PCR, and Western blot showed strong upregulation of TRIB2 protein levels in enzalutamide-resistant (LNCaP-ENR, PCa-2B-ENR, and LAPC4-ENR) prostate cancer cells (Fig. 1, *E* and *F*). Overexpression of TRIB2 is correlated with activation of the canonical Akt-signaling module showing increased phosphorylation of Akt (pSer-473) and increased protein level of Bcl-xL, which are standard markers that promote cell survival and decrease apoptosis in a variety of cells. Consistent with our results, we also found elevated mRNA levels of TRIB2 in gene expression datasets in recognized enzalutamide-resistant prostate cancer cells (Fig. S1) (16). Enzalutamide-resistant cells also showed increased levels of TRIB2 protein compared to enzalutamide-sensitive cells (Fig. S2). Increased mRNA expression of TRIB2 (denoted as GS3955) was observed previously by Bisoffi *et al.* (17), in the androgen-resistant (C4-2 and PC3) prostate cancer cells compared to androgen-sensitive (LNCaP) cells.

To verify whether the *in vitro* observation of TRIB2 overexpression is valid *in vivo*, we comprehensively analyzed prostate tumors in tissue microarrays (TMAs). We found that patient-derived xenografts of prostate tumors overexpress TRIB2 when the mice were treated with enzalutamide at 30 mg/kg/day for 6 weeks (Fig. 1, *G* and *H*). Moreover, we found that a vast majority of the clinically advanced metastatic prostate tumors from enzalutamide-treated patients show a robust increase in the expression of TRIB2 proteins (Fig. 1, *I* and *J*). Altogether, these findings suggest that triggering of TRIB2 overexpression is a fundamental mechanism in prostate cancer cells both *in vitro* and *in vivo* when treated with enzalutamide, a second-generation direct inhibitor of AR activity. *Note*: To confirm the specificity of TRIB2 antibodies, we used a panel of TRIB2-negative and TRIB2-positive cell lines and tumor tissues and analyzed by both Western blot as well as immunohistochemistry (Fig. S3).

### TRIB2 plays a critical role in enzalutamide-resistant prostate cancer cells

**Figure 2:**
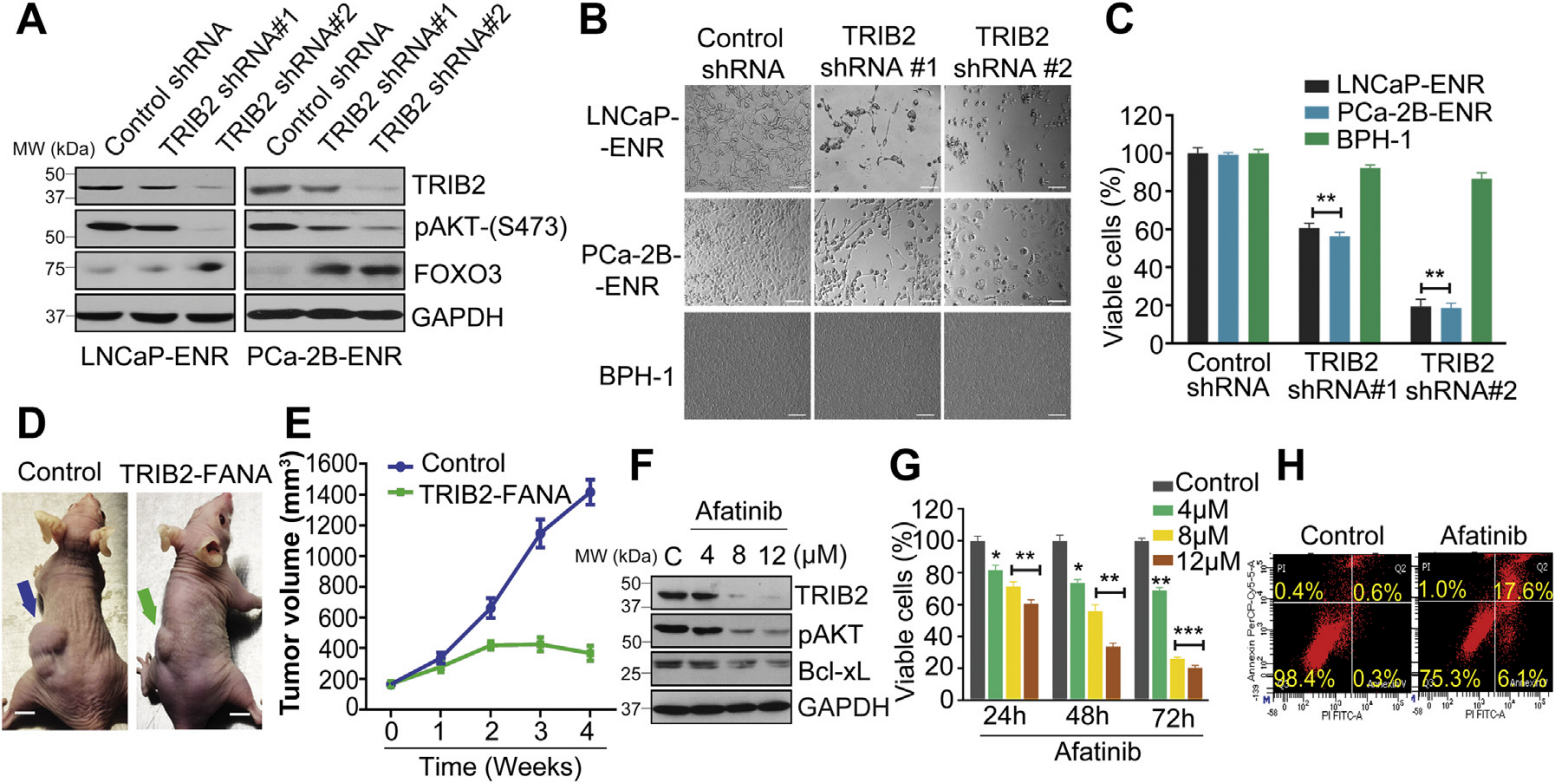
**Inhibition of TRIB2 killsenzalutamide-resistantprostate cancer cells via induction of apoptosis.***A*, cells were plated overnight and treated with gene-specific shRNAs for 4 days. Protein levels of TRIB2, p-Akt, and forkhead box O3-alpha (FOXO3a) were detected by Western blot. *B* and *C*, morphological alterations and viability of cells were detected 4 days after shRNA treatment (figure *B*, the scale bar represents 400 μm). *D* and *E*, effect of FANA-modified TRIB2 antisense oligos on LNCaP-ENR tumor growth was tested in nude mice xenografts (n = 3) by intratumoral delivery at 2 mg/kg/day every fourth day. Representative images of tumor-bearing mice from different groups were taken at the end of the study (the scale bar represents 10 mm). The tumor volumes were measured by vernier calipers and presented as mean values ± SE. *F*, LNCaP-ENR cells were treated with Afatinib (AFA) for 24 h and protein levels were detected by Western blot. *G*, time-dependent decrease in the viability of LNCaP-ENR cells was measured by MTS/PES assay. *H*, apoptosis was measured by Annexin V binding after treating LNCaP-ENR cells with 12 μM AFA for 24 h. Positions of the molecular weight markers for the ladder are indicated along with the Western blot data. The data are presented as mean values ± SE. ∗*p* < 0.05; ∗∗*p* < 0.005; ∗∗∗*p* < 0.0005. For *C* and *G*, Two-way ANOVA, Tukey’s multiple-comparison test was applied. FANA, 2'-deoxy-2'-fluoro-beta-D-arabinonucleic acid; TRIB2, Tribbles 2.

Because overexpression of TRIB2 was observed in prostate cancer cells upon enzalutamide treatment both *in vitro* and *in vivo*, we asked the question whether TRIB2 plays any role in enzalutamide-resistant cells. By shRNA-mediated knockdown we found that downregulation of TRIB2 strongly inhibits the viability and growth of enzalutamide-resistant cells, whereas benign prostatic hyperplasia (BPH-1) cells remained unaffected (Figs. 2, *A*–*C* and S4), suggesting that TRIB2 plays a critical but selective role in enzalutamide-resistant prostate cancer cells. We also found that TRIB2 siRNA decreased LNCaP-ENR tumor growth in nude mice (Fig. 2, *D* and *E*). No specific, target-validated inhibitor of TRIB2 is commercially available. However, recently, it was demonstrated that the EGFR kinase inhibitor, Afatinib (AFA), destabilizes TRIB2 protein by covalent modification and primes TRIB2 for degradation at high micromolar doses (18). Thus, we also examined the effect of AFA and found that it strongly downregulates TRIB2 protein level and kills enzalutamide-resistant prostate cancer cells by triggering apoptosis (Fig. 2, *F*–*H*). These findings indicate that TRIB2 plays an important role in enzalutamide-resistant cells and suggest that suitable more selective TRIB2-targeting agents could be developed to kill enzalutamide-resistant prostate cancer cells.

### TRIB2 enhances prostate cancer cell growth and invasion and confers resistance to enzalutamide

**Figure 3:**
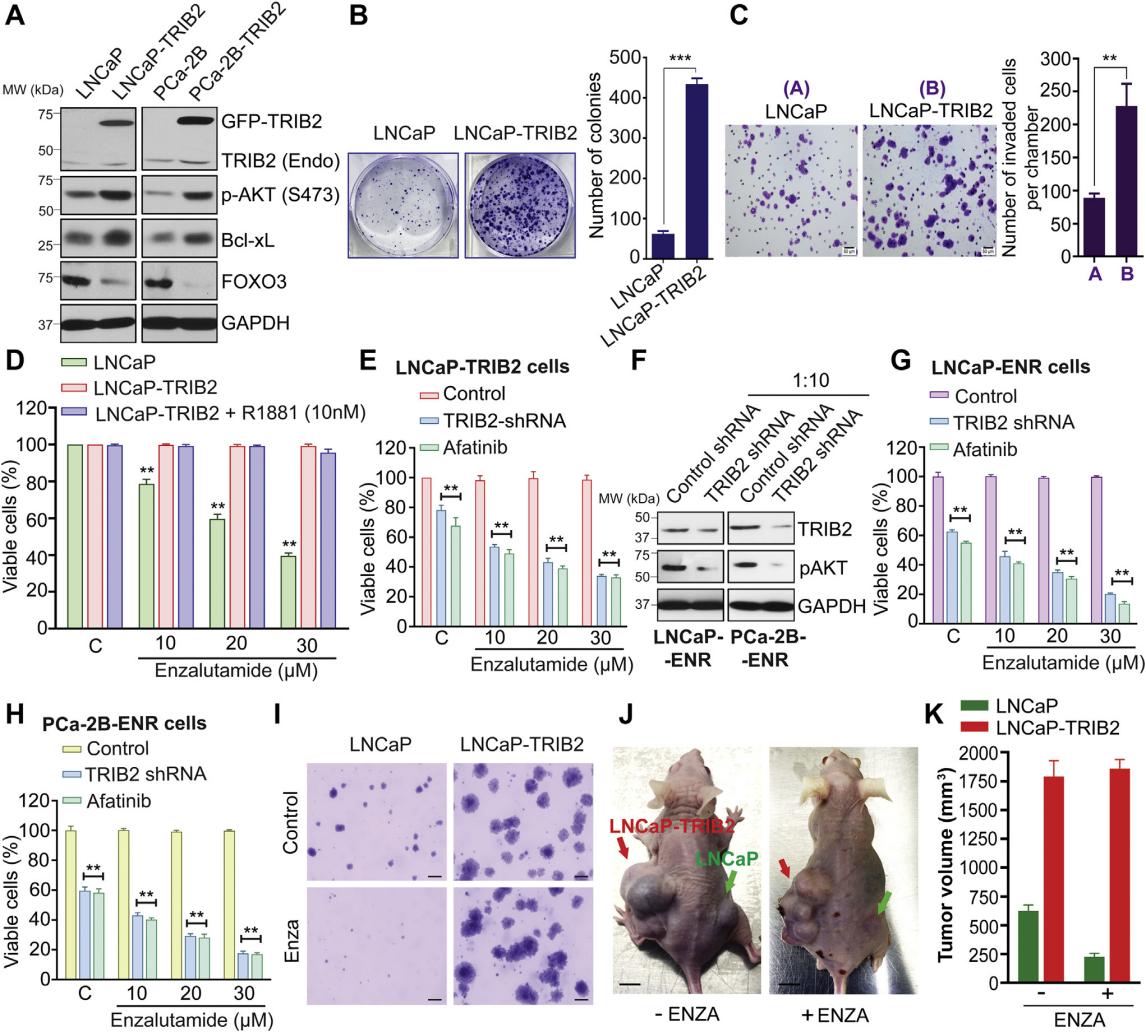
**TRIB2 enhances prostate cancer cell growth and invasion and confers resistance to enzalutamide.***A*, immunoblot analysis showing the levels of selected proteins in TRIB2-OE cells. *B*, colony growth of transfected and parental cells were analyzed after staining with 0.025% crystal violet. *C*, *in vitro* invasion was measured using Matrigel-coated Boyden chambers (the scale bar represents 50 μm). *D*–*H*, sensitivity of parental and TRIB2-OE cells to enzalutamide was measured by MTS/PES assay. The role of TRIB2 in enzalutamide resistance was verified by treating LNCaP-TRIB2 cells (*E*) and ENR cells (*F*–*H*) with shRNA (1:10) or by Afatinib (6 μM). Positions of the molecular weight markers for the ladder are indicated along with the Western blot data. *I*–*K*, impact of TRIB2 overexpression on enzalutamide resistance was measured in soft-agar colony formation assay and in nude mice giving 30 mg/kg/day for 4 weeks (n = 3) (*I*, the scale bar represents 20 μm). Representative pictures of tumor-bearing mice from different groups as indicated were taken at the end of the study (the scale bar represents 10 mm). Tumor volumes were measured by vernier calipers and presented as mean values ± SE. The data presented as mean values ± SE. ∗∗*p* < 0.005; ∗∗∗*p* < 0.0005. For *D*, *E*, *G*, and *H*, Two-way ANOVA, Tukey’s multiple-comparison test was applied. TRIB2, Tribbles 2; TRIB2-OE, TRIB2-overexpressing.

Because TRIB2 plays a critical role in the viability of enzalutamide-resistant prostate cancer cells, it has emerged as a new molecular target for therapeutic development. It also raised the question whether overexpression of TRIB2 provides any growth advantage or plays an active role in resistant prostate cancer cells for their defense against enzalutamide therapy. To address this, we transfected LNCaP and PCa-2B cells with full-length human TRIB2 gene and found that the TRIB2-overexpressing (TRIB2-OE) cells (LNCaP-TRIB2 and PCa-2B-TRIB2) show increased levels of prosurvival proteins, p-Akt and Bcl-xL, and decreased level of Forkhead Box O3, a tumor suppressor (Fig. 3*A*). TRIB2-OE cells also showed increased number and size of colonies (Fig. 3*B*) and increased capability to invade through Matrigel in Boyden chambers (Fig. 3*C*), suggesting that TRIB2 may play a major role as a driver to promote aggressive behavior which is frequently observed in antiandrogenic therapy-resistant prostate cancer cells. We also found that overexpression of TRIB2 alone makes prostate cancer cells resistant to therapeutic doses of enzalutamide, and the enzalutamide-resistant cells are not sensitive to the synthetic androgen, R1881 (Figs. 3*D* and S5). Interestingly, this resistance is abolished, and the TRIB2-OE cells become sensitive to enzalutamide again when treated with TRIB2-shRNA or AFA (Fig. 3*E*). Similar resensitization to enzalutamide was also observed in LNCaP-ENR and PCa-2B-ENR cells when TRIB2 was inhibited (Fig. 3, *F*–*H*). We also found that the naturally occurring castration-resistant prostate cancer cells, C4-2B, reverted to enzalutamide-sensitive state upon TRIB2 knockdown (Fig. S6). TRIB2-OE cells form larger soft-agar colonies and show no sensitivity to enzalutamide (Fig. 3*I*). Furthermore, we found that overexpression of TRIB2 enhances prostate tumor growth in nude mice and these tumors grow uninterrupted with enzalutamide treatment (Fig. 3, *J* and *K*). These findings suggest that not only TRIB2 enhances the aggressive characteristics, but also greatly contributes to the enzalutamide resistance mechanism in prostate cancer cells.

### TRIB2 confers resistance to enzalutamide by promoting lineage plasticity to develop NE phenotype

**Figure 4:**
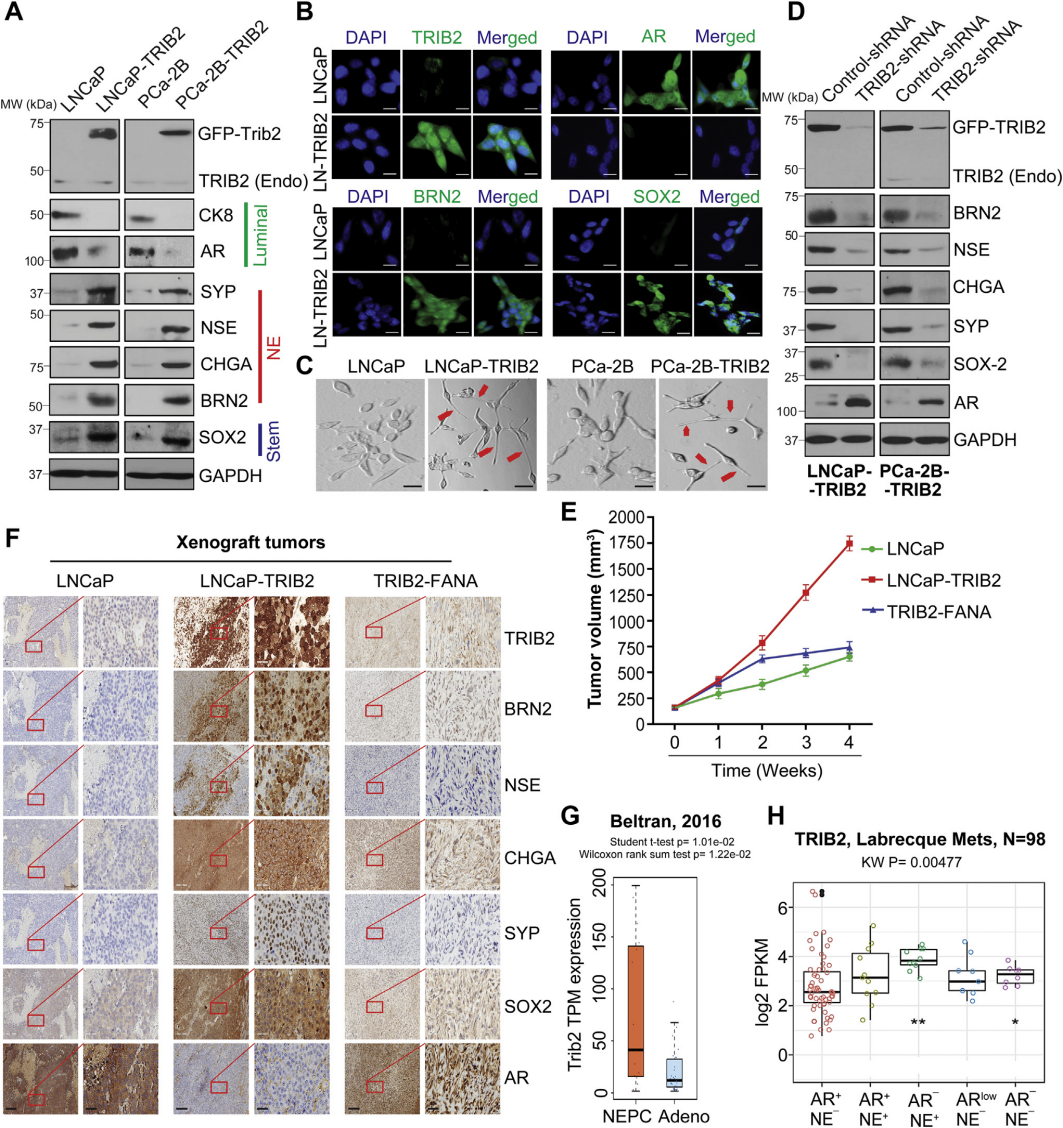
**TRIB2 confers resistance to enzalutamide by promoting lineage plasticity.***A* and *B*, Western blot and immunofluorescence analysis showing protein levels of NE (BRN2) and stemness (SOX2) markers in TRIB2-OE cells, respectively (*B*, the scale bar represents 10 μm). *C*, phase contrast microscope pictures showing small cells with well-developed needle like processes or neurites (*red arrows*) (the scale bar represents 400 μm). *D*, immunoblot for NE and stemness markers in TRIB2-shRNA treated cells. Positions of the molecular weight markers for the protein ladder are indicated along with the Western blot data. *E*, effect of FANA-modified TRIB2 antisense oligos on LNCaP-TRIB2 tumor growth was tested in nude mice xenografts (n = 3) by intratumoral delivery at 2 mg/kg/day every fourth day. Tumor volumes were measured by vernier calipers and presented as mean values ± SE. *F*, immunohistochemical (IHC) analysis showing NE and stemness markers in LNCaP, LNCaP-TRIB2, and TRIB2-FANA xenograft tumors (the scale bar represents 200 μm; the scale bar represents 50 μm (*inset*)). *G*, boxplot showing TRIB2 expression in neuroendocrine prostate cancer (NEPC) *versus* prostate adenocarcinoma (Adeno) patients (8). Wilcoxon rank sum test was used to test the difference between the two groups. *H*, TRIB2 expression in prostate cancer phenotypes (9). AR-high prostate cancer (AR^+^/NE^–^), Amphicrine prostate cancer (AR^+^/NE^+^), neuroendocrine prostate cancer (AR^–^/NE^+^), AR-low prostate cancer (AR-low/NE^–^), and double-negative prostate cancer (AR^–^/NE^–^). The results are expressed as log2 fragments per kilobase of transcript per million mapped reads (FPKM). The data presented as mean values ± SE. ∗*p* <0.05; ∗∗*p* < 0.005. For E and F, Two-way ANOVA, Tukey’s multiple-comparison test was applied. BRN2, Brain-2; FANA, 2'-deoxy-2'-fluoro-beta-D-arabinonucleic acid; NE, neuroendocrine; SOX2, SRY-box 2; TRIB2, Tribbles 2; TRIB2-OE, TRIB2-overexpressing.

How TRIB2 confers resistance to enzalutamide is an intriguing question. Resistance to androgen-signaling blockers may happen because of the overexpression or mutation of the AR gene, or due to development of mechanism(s) independent of androgenic signaling. To address the downstream mechanism of TRIB2 action in enzalutamide resistance, we found that TRIB2-OE enzalutamide-resistant cells (LNCaP-TRIB2 and PCa-2B-TRIB2) show a decrease in the protein level of luminal markers (cytokeratin 8 (CK8) and AR), but there is a strong increase in the expression levels of NE markers, such as Synaptophysin (SYP), Enolase-2 (NSE), and Chromogranin-A (CHGA) (Fig. 4, *A* and *B*). These findings indicated that TRIB2-induced resistance to enzalutamide does not involve reactivation of the AR function or maintenance of the luminal features, rather TRIB2 appears to be involved in transdifferentiation of luminal epithelial cells to develop NE-like characteristics (Fig. 4*C*). Transcriptomic analysis of enzalutamide-resistant prostate cancer cells by us and others (16) also revealed a negative correlation between the expression of TRIB2 and AR signaling-associated genes (Fig. S7).

Notably, we found a strong upregulation of the neuronal transcription factor, BRN2, and the stemness transcription factor, SOX2, in TRIB2-OE enzalutamide-resistant cells (Figs. 4, *A* and *B* and S8), which were characterized to promote lineage plasticity in prostate cancer cells (16, 19). The regulation of NE and stemness markers by TRIB2 was confirmed by shRNA knockdown of TRIB2 in TRIB2-OE cells (Fig. 4*D*). We also found overexpression of NE markers and downregulation of AR in TRIB2-OE cells in tumor xenografts in mice (Fig. 4, *E* and *F*). Moreover, we found consistent strong expression of TRIB2 in multiple standard NE-type prostate tumor samples (Figs. S9 and S10). Both the Beltran and Labrecque databases show overexpression of TRIB2 in NE-type prostate cancers compared to adenocarcinoma (Fig. 4, *G* and *H*). Interestingly, inhibition of either BRN2 or SOX2 resensitizes TRIB2-OE cells to enzalutamide treatment, indicating that the molecular mechanism of TRIB2 may involve upregulation of BRN2 and SOX2, presumably to increase cellular plasticity (Fig. S11). Altogether, our findings suggest that TRIB2 helps prostate cancer cells to evade enzalutamide therapy, apparently by switching their identity from luminal to assume NE characteristics. Thus, it appears that a strong positive correlation exists between TRIB2 overexpression and the development of NE features in prostate cancer cells.

## Discussion

Targeting of androgenic signaling has been improved significantly due to the introduction of strong AR antagonists, though the benefit is temporary, and resistance invariably develops which mostly occurs within 5 years since therapy begins (5, 6, 7). It has been realized that enzalutamide-resistant prostate cancer frequently assumes deadly phenotype if AR-independent mechanisms develop, though the spectrum of which is yet to be fully characterized. We observed that overexpression of TRIB2 occurs upon treatment with enzalutamide, both *in vitro* and *in vivo* (Fig. 1). Our findings of the gross overexpression of TRIB2 by enzalutamide treatment and the aggressive growth characteristics of TRIB2-OE cells signify a negative impact of AR blockade therapy for prostate cancer. Though upregulation of TRIB2 in prostate cancer cells upon enzalutamide treatment is a remarkable finding, why and how TRIB2 is overexpressed in these cells is not known at this time. Of the multitude of factors playing roles in enzalutamide resistance, derepression of AR-controlled suppression of gene expression seems to be one. We found that PC3 and DU145 prostate cancer cells that are naturally deficient in AR activity express high levels of TRIB2 protein. Interestingly, forced overexpression and activation of full-length AR downregulates TRIB2 protein level, suggesting a negative regulation of TRIB2 by AR (Fig. S12). Negative regulation of the neuronal transcription factor (BRN2) and SPINK by AR has been found recently (16, 20). The aggressive nature of prostate cancer cells post-enzalutamide therapy, correlates well with aggressive melanoma and lung cancer cells which overexpress TRIB2. This could be, at least in part, because of the absence of strong androgenic signaling in skin and lung cells, the presence of which may inhibit the expression of TRIB2 in prostate cells. However, further work is needed to substantiate this notion.

Curiosity lingered around the consequence of TRIB2 overexpression in prostate cancer cells upon enzalutamide treatment. TRIB2 was originally discovered in *Drosophila* as a regulator of wing pattern (21). Later, its expression in human and other organisms as well as a role in cancer cells were observed (22, 23). We wanted to address whether TRIB2 plays any role in enzalutamide-resistant prostate cancer cells. Interestingly, we observed that treatment with TRIB2-shRNA decreased TRIB2 protein level and proportionately reduced the viability of enzalutamide-resistant cells, suggesting that TRIB2 plays a critical role in the enzalutamide resistance mechanism in prostate cancer cells (Fig. 2). That overexpression of TRIB2 alone can confer complete resistance to physiological doses of enzalutamide is particularly interesting (Fig. 3). However, a more remarkable feature recently revealed from our work, is the resistance of TRIB2-OE prostate cancer cells also to other second-generation anti-androgens that are frequently used in the clinic in addition to enzalutamide, such as apalutamide, darolutamide, and abiraterone (not shown).

Intriguingly, enzalutamide-resistant TRIB2-OE cells show decreased protein level of luminal markers (CK8 and AR) and increased the level of NE markers (BRN2, SYP, NSE, and CHGA), and these characteristics are reversed when TRIB2 is downregulated by shRNA (Fig. 4, *A* and *D*). These findings suggest that TRIB2 is a driver for transdifferentiation of prostate cancer cells from luminal to NE type. An AR activity low stemness program as well as NE differentiation were observed in enzalutamide-resistant prostate cancer cells and tumors (8, 9, 10, 11, 12, 24, 25). Recently, the SOX2-mediated lineage plasticity and development of NE phenotype have been demonstrated in PTEN ^(−/−)^: Rb1 ^(−/−)^ double knockout mice (19, 26). Moreover, a role of BRN2 was documented in treatment-emergent NE differentiation via upregulation of SOX2 (16). We found that the overexpression of TRIB2 strongly upregulates BRN2 and SOX2. Moreover, inhibition of either BRN2 or SOX2 resensitizes TRIB2-OE cells to enzalutamide treatment, suggesting that the NE characteristics may play an important role in enzalutamide resistance. Abrupt deviation of cellular characteristics is an indication that the molecular mechanism of TRIB2 involves upregulation of BRN2 and SOX2, presumably to increase cellular plasticity and lineage switching.

Based on our findings, it appears that TRIB2 induces lineage switching by developing stemness and NE characteristics, which supports enzalutamide-resistant prostate cancer cells in a way that they are no longer required to depend on AR-mediated signaling. Development of AR (-)/low tumors have now been found to occur in as high as 36% of the patients when treated with anti-androgens, such as enzalutamide, and this rate is expected to go up with stronger agents to eliminate the AR function altogether (27, 28). We also found a strong positive correlation of TRIB2 in a range of NE-type prostate tumor samples (Figs. S9 and S10). Notably, we observed that the overexpression of TRIB2 promotes NE features in prostate cancer cells, and this is reversed with inhibition of TRIB2. Thus, TRIB2 emerges as a new driver (rather than just a biomarker) that helps prostate cancer cells to evade enzalutamide therapy, apparently by switching their identity from the parental luminal type and developing NE characteristics. TRIB2 is known to regulate the *String* and *Twine* genes and plays a role in *Drosophila* morphogenesis (21). TRIB2 was also characterized to activate Akt and ERK via a still obscure mechanism to support aggressive cancer growth and therapeutic resistance (22, 23). From our unprecedented observations in prostate cancer, it became apparent that TRIB2 is a *bona fide* driver for enhanced growth and enzalutamide resistance and works via a mechanism involving the promotion of lineage plasticity and transdifferentiation so that prostate cancer cells can overcome the loss of support caused by interruption of the androgenic signaling axis.

## Experimental procedures

### Cell lines and tissue culture

LNCaP, MDA-PCa-2B, LAPC4 human prostate cancer cell lines and benign prostatic hyperplasia epithelial cell line, BPH-1, were purchased from American Type Culture Collection. The enzalutamide-resistant LNCaP-ENR, MDA-PCa-2B-ENR, and LAPC4-ENR cells were generated by treating with the increasing concentrations of enzalutamide over 3 months. The castration-resistant 16D^CRPC^ and enzalutamide-resistant 42D^ENZR^, 49F^ENZR^, and 49C^ENZR^ cells were kindly provided by Dr Amina Zoubeidi (Vancouver Prostate Center). The cells were grown in RPMI medium 1640 or DMEM (Invitrogen) or HPC1 media (Athena Enzyme). All the media were supplemented with 10% fetal bovine serum and antibiotics. All the enzalutamide-resistant cells were cultured in the presence of 30 μM enzalutamide.

### RNA sequencing and analysis

Total RNA was isolated from exponentially growing cells using spin columns and reagents in the RNeasy Midi Kit following methods provided by the company (Qiagen). RNA quality check, cDNA synthesis, and sequencing were performed at the AGTC core facility using the Illumina Hi-Seq platform. The analysis of differentially expressed genes between groups was conducted using a negative binomial model as implemented in the EdgeR package in R. A gene with a fold-change > 2 (or < 0.5) and a False Discovery Rate-adjusted *p*-value < 0.05 is considered differentially expressed between two experimental conditions. The heatmap of differentially expressed genes was plotted using the gplots heatmap.2 function in R. Each row was scaled so that it has a mean of zero and a SD of one. The volcano plot showing the differentially expressed gene results was generated using the EnhancedVolcano package in R.

### Real-time quantitative PCR

Total RNA was extracted using RNeasy kit (Qiagen), and 1 μg of total RNA was used for cDNA synthesis using SuperScript III First-Strand kit (Invitrogen) according to the manufacturer’s instructions. PCR reaction mixture was prepared using gene-specific TaqMan gene expression assay system (Applied Biosystems). qRT-PCR reactions were performed in triplicate using QuantStudio six Flex Real-Time fast PCR System (Applied Biosystems), and 2^−ΔΔCt^ values were used to calculate the relative expression level of the target genes compared to controls. GAPDH was used as a normalization control.

### Western blot

Cells were plated in 60 mm diameter plates and at 60 to 70% confluency, they were treated with inhibitors. After treatment, the cells were harvested, washed, and lysed in lysis buffer (50 mM Hepes buffer, pH 7.4, 150 mM NaCl, 1 mM EDTA, 1 mM orthovanadate, 10 mM sodium pyrophosphate, 10 mM sodium fluoride, 1% NP-40, and a cocktail of protease inhibitors). Proteins were separated by 12% SDS–PAGE and transferred to nitrocellulose membranes. The membranes were blocked with 5% nonfat-milk solution and blotted with appropriate primary antibody followed by peroxidase-labeled secondary antibody. The bands were visualized by enhanced chemiluminescence detection kit from Pierce Biotech. To be accepted as valid, protein blots were analyzed at least in two independent experiments showing similar results. Antibodies against TRIB2, p-AKT, Bcl-xL, AR, PSA, Nkx3.1, SOX2, and GAPDH were from Santa Cruz Biotechnology. Antibodies against BRN2, NSE, SYP, CHGA, and CK8 were from Cell Signaling Technology.

### Human prostate cancer specimens and patient-derived xenograft tumors

Tissue microarray with enzalutamide-treated prostate cancer specimens were obtained from the Prostate Cancer Biorepository Network, University of Washington, US Biomax, Inc, and Cybrdi Inc, University of Michigan. The hormone naïve prostate tumor TMAs were obtained from Henry Ford Health System biorepository after mandatory approval from the Institutional Review Committee. TMA slides created from PDX tumors (Untreated and enzalutamide treated) were obtained from Dr Martin E. Gleave, Vancouver Prostate Centre.

### Immunohistochemistry

Slides containing formalin-fixed paraffin-embedded sections were incubated at 60 °C for 1 h, and antigen retrieval was carried out in EnVision FLEX target retrieval solution, Low pH (Agilent Dako, S236984–2) in a PT Link instrument (Agilent Dako, PT200). The slides were washed in 1X TBST wash buffer for 5 min, followed by treatment with Peroxidazed 1 (Biocare Medical, PX968 M) for 5 min. Nonspecific background was blocked with Background punisher (Biocare Medical, BP974) with a 10 min treatment. The slides were incubated with appropriate primary antibodies diluted in EnVision FLEX Antibody Diluent (Agilent Dako, K800621–2) overnight at 4 °C. The slides were washed and incubated with Mach2 Double Stain one or 2 (Biocare Medical, MRCT523/525) for 30 min at room temperature. The Slides were developed using ImmPACT DAB Substrate, Peroxidase (HRP) (Vector Labs, SK-4105) and counterstained with Hematoxylin (Agilent DAKO, K800821–2) for 5 min. The slides were rinsed in distilled water, dried, and mounted using EcoMount (Biocare Medical, EM897 L). The slides were scanned using Aperio CS2 digital pathology scanner, and the staining intensity was quantified by QuPath 0.2.3 (Github) digital image analysis software.

### Plasmids and lentiparticles

TRIB2-GFP plasmids were obtained as a kind gift from Dr Wolfgang Link. Mission lentiviral transduction particles for TRIB2-shRNA and the siRNAs targeting BRN2 and SOX2 were purchased from Sigma Aldrich. For transfection of siRNAs, Lipofectamine RNAiMAX was used in accordance with the manufacturer's guidelines (Life Technologies).

### Cell viability assay

The cells (∼3000 per well) were plated overnight in 96-well tissue culture plates and treated with drugs or appropriate controls. After 72 h, cell viability was measured by MTS/PES One Solution Cell Titer Assay following manufacturer’s protocol (Promega Corp).

### Soft-agar colony formation assay

LNCaP and LNCaP-TRIB2 cells (1 × 10^5^) were mixed in 0.5 ml of 0.3% soft-agar and seeded on the top of a 2 ml base layer of 0.6% agar. Plates were allowed to settle and then the agar layers were covered with 2 ml fresh RPMI media containing 10% fetal bovine serum. The cells were treated with enzalutamide (30 μM) for 3 weeks. Cell growth medium and enzalutamide were exchanged every fourth day. At the end of incubation, the cells were stained with 0.025% crystal violet, and the colonies were counted and photographed under a Leica microscope at ×150.

### *In vivo* tumor models

Animal studies were approved by the Institutional Animal Care and Use Committee and performed according to the institutional guidelines for animal care and handling. To analyze the effect of TRIB2 knockdown on the growth on prostate tumors *in vivo*, exponentially growing LNCaP-ENR cells (3 × 10^6^ cells/mouse in 50% Matrigel in PBS) were subcutaneously injected into the flanks of 7-week-old male athymic nude mice (*n* = 3). When the tumors grew to approximately 100 mm^3^, mice were randomized and treated either with control or TRIB2 FANA (2'-deoxy-2'-fluoro-beta-D-arabinonucleic acid) oligos from Aum Biotech via intratumoral injection (2 mg/kg/day every fourth day) for 4 weeks. To study the impact of TRIB2 overexpression on enzalutamide resistance *in vivo*, LNCaP or LNCaP-TRIB2 cells (3 × 10^6^ cells/site) were implanted subcutaneously into the right or left flanks of nude mice (*n* = 3) and allowed to grow until the tumors were palpable. The mice were randomized to receive either vehicle (DMSO alone) or enzalutamide (30 mg/kg/day) for 4 weeks. Tumor growth was monitored by measuring volumes using a digital slide-calipers.

### Statistical analysis

Statistical significance was assessed by either one-way ANOVA or two-way ANOVA with post hoc multiple comparisons test or the two-tailed Student’s *t* test using GraphPad prism software. The *p*-value of less than 0.05 was defined as significant. The results are expressed as the mean ± SEM from at least three independent experiments and are described in each figure legend when applied.

## Data availability

All the data are available either in the main Manuscript or in the Supporting Document.

## Supporting information

This article contains supporting information (16, 26).

## Conflict of interest

The authors declare that they have no conflicts of interest with the contents of this article.
