# Supplementary material for: Tribbles 2 pseudokinase confers enzalutamide resistance in prostate cancer by promoting lineage plasticity
Source: J Biol Chem. 2021 Dec 30;298(2):101556. doi: 10.1016/j.jbc.2021.101556 (PMC8800106; doi:10.1016/j.jbc.2021.101556)
Supplement: Supplemental Figures S1–S12 [file mmc1.pdf]

Supplementary Materials for

Tribbles 2 pseudokinase confers enzalutamide resistance in prostate cancer by promoting lineage plasticity

Authors: Jitender Monga[1], Indra Adrianto[2], Craig Rogers[1,4], Shirish Gadgeel[4], Dhananjay Chitale[3,4,] Joshi J. Alumkal[5], Himisha Beltran[6], Amina Zoubeidi[7], and Jagadananda Ghosh[1,4,*]

Affiliations:

[1]Vattikuti Urology Institute, Henry Ford Health System, Detroit, MI 48202, USA.

[2]Public Health Sciences, Henry Ford Health System, Detroit, MI 48202, USA.

[3]Department of Pathology, Henry Ford Health System, Detroit, MI 48202, USA.

[4]Henry Ford Cancer Institute, Henry Ford Health System, Detroit, MI 48202, USA.

[5]Univeristy of Michigan, Ann Arbor, MI 48108, USA.

[6] Dana Farber Cancer Institute and Harvard Medical School, Boston, MA 02115, USA.

[7] Vancouver Prostate Center, Vancouver, BC, Canada.

*Correspondence to: jghosh1@hfhs.org

This PDF file includes:

Figs. S1 to S12

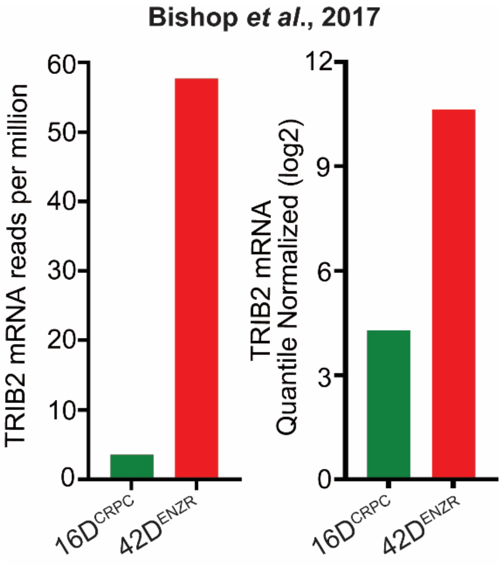

Fig. S1. RNA-seq datasets of TRIB2 in enzalutamide resistant prostate cancer cells. mRNA expression profiles from RNA-seq datasets (Bishop *et al*., 2017; Ref: 16) showing elevated level of TRIB2 in enzalutamide resistant cell line ($42D^{ENZR}$).

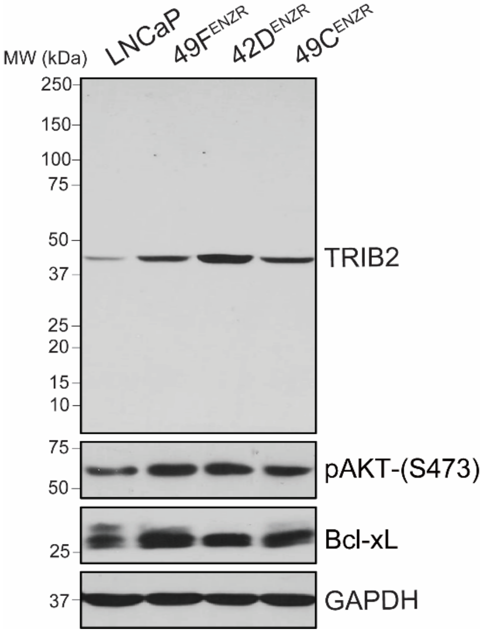

Fig. S2. Enzalutamide resistant prostate cancer cells show higher levels of TRIB2. Western blot showing increased protein levels of TRIB2 and targets in enzalutamide-resistant prostate cancer cells, compared to parental enzalutamide-sensitive cells. Positions of the molecular weight markers for the ladder are indicated along with the western blot data.

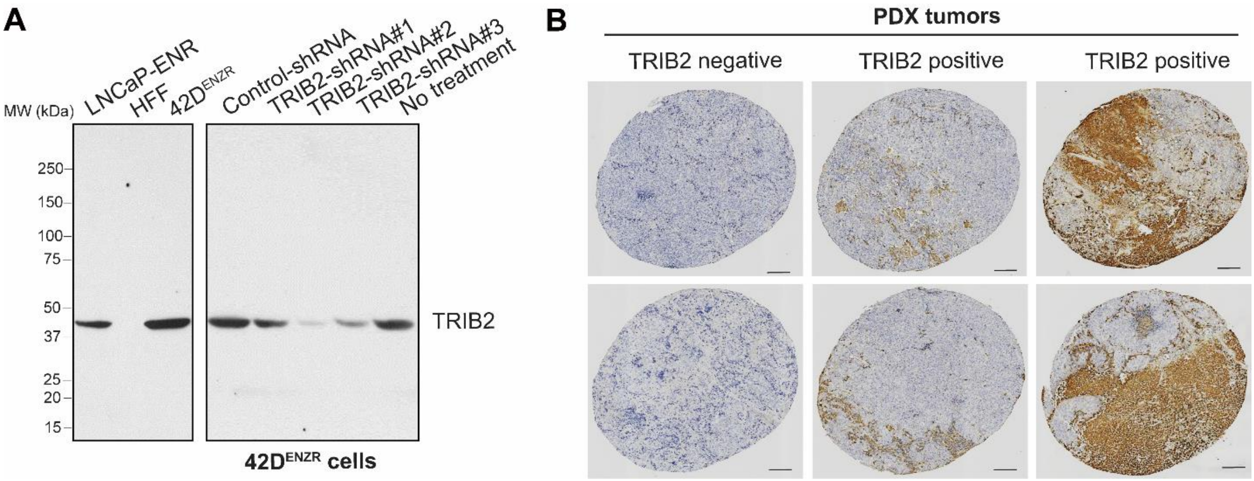

Fig. S3. TRIB2 antibody validation data. A) Representative Western blot images of TRIB2-low, TRIB2-High and TRIB2-KD prostate cancer cells. Western blot was performed with monoclonal antibody from Santa Cruz Biotech (Mouse monoclonal, Catalog # sc-100878). ***Note:*** Single band in Western blot is a consistent observation with monoclonal antibodies from both Santa Cruz Biotech (Mouse monoclonal, Catalog # sc-100878) and Cell Signaling Technology (Rabbit monoclonal, Catalog #13533). Positions of the molecular weight markers for the ladder are indicated along with the western blot data. **B)** IHC images of TRIB2-negative and TRIB2-positive prostate PDX tumors stained with Cell Signaling Technology (Rabbit monoclonal, Catalog #13533) (Scale bar = 200μm). TRIB2 positive IHC pictures are reused from Fig. 1G as a representation of antibody validation.

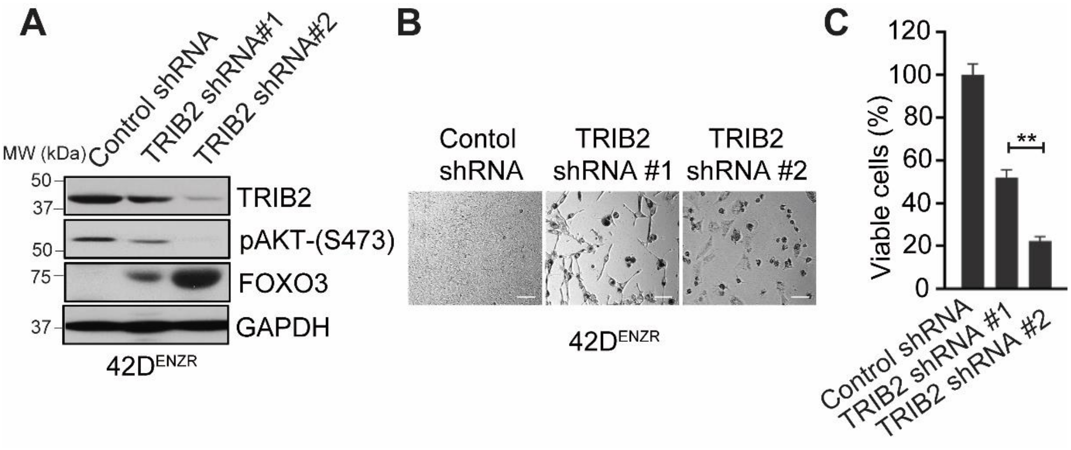

Fig. S4. Inhibition of TRIB2 kills enzalutamide-resistant prostate cancer. A) Cells were plated overnight and treated with TRIB2 specific shRNAs for four days. Protein levels of indicated proteins were detected by Western blot. Positions of the molecular weight markers for the ladder are indicated along with the western blot data. **B, C)** Morphological alterations and viability of cells were detected four days after shRNA treatment (*Panel B:* Scale bar = 400μm). Data presented as mean values ± SE. ***p*<0.005. For panels C, Two-way ANOVA, Tukey's multiple-comparison test was applied.

.

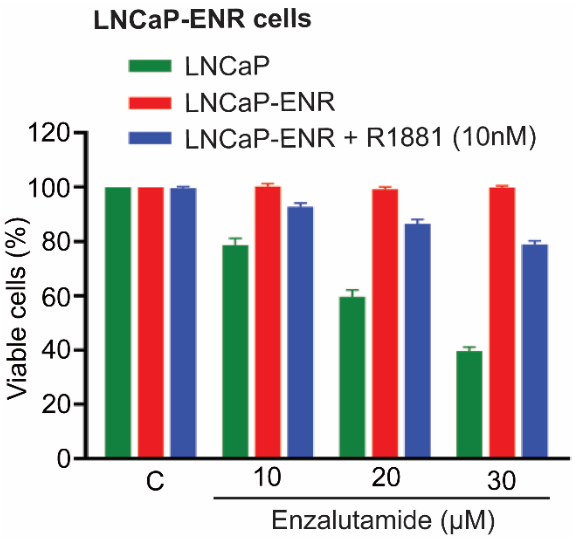

Fig. S5. Sensitivity of enzalutamide resistant cells to synthetic androgen. Indicated cells were treated with R1881 and/or enzalutamide for 72 hours and cell viability was measured by MTS/PES assay.

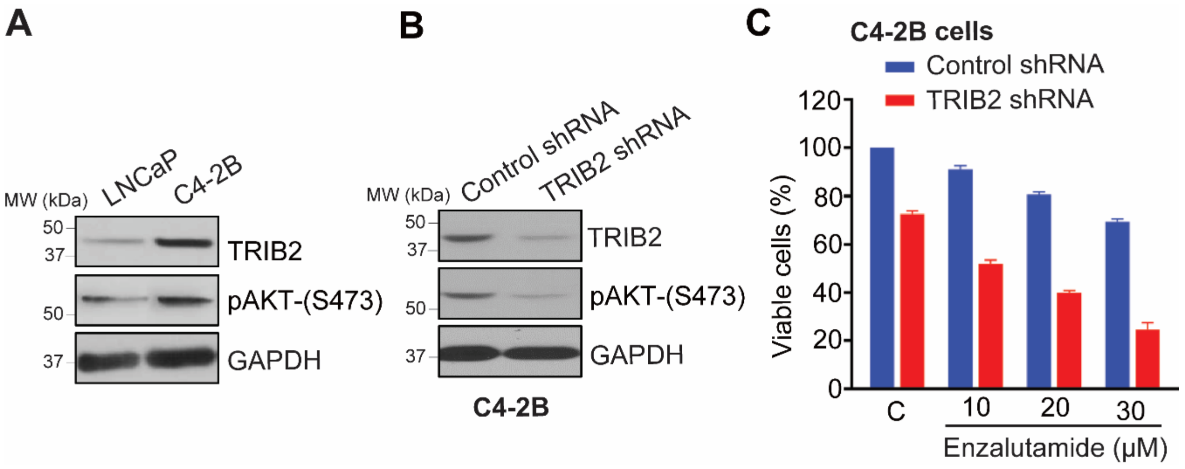

Fig. S6. TRIB2 inhibition re-sensitizes CRPC cells to enzalutamide. A) Immunoblot analysis showing TRIB2 protein expression levels in androgen-sensitive and androgen-insensitive prostate cancer cells. **B)** Protein levels of selected proteins upon TRIB2 knockdown in CRPC cells. Positions of the molecular weight markers for the ladder are indicated along with the western blot data. **C)** Effects of TRIB2 knockdown on the cell viability of CRPC cells treated with enzalutamide was measured by MTS assay.

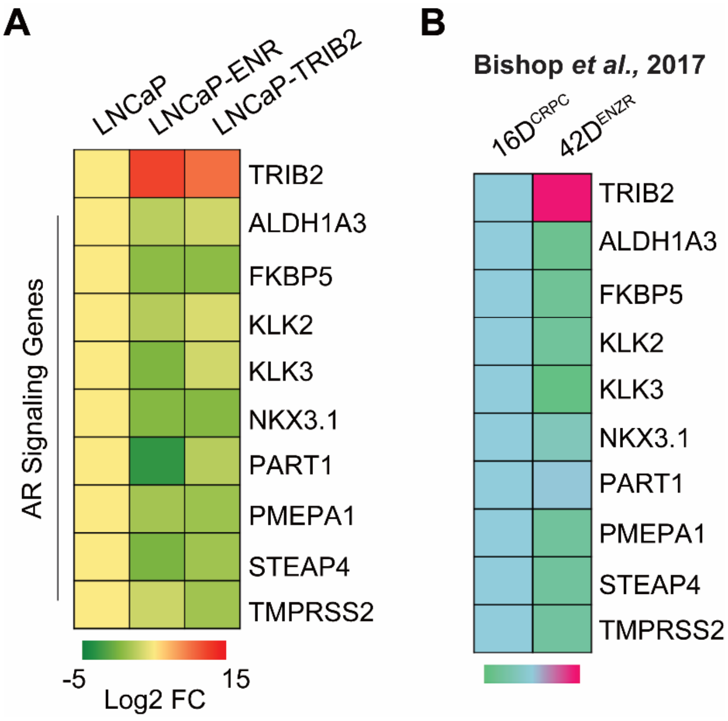

Fig. S7. Enzalutamide resistant prostate cancer cells show high levels of TRIB2 expression and low levels of AR signaling markers. A, B) Heatmaps showing fold increase in TRIB2 gene versus AR target genes in enzalutamide resistant prostate cancer cell models. ***Note*:** Overexpression of TRIB2 was found to be associated with low AR function in multiple prostate cancer cell culture models.

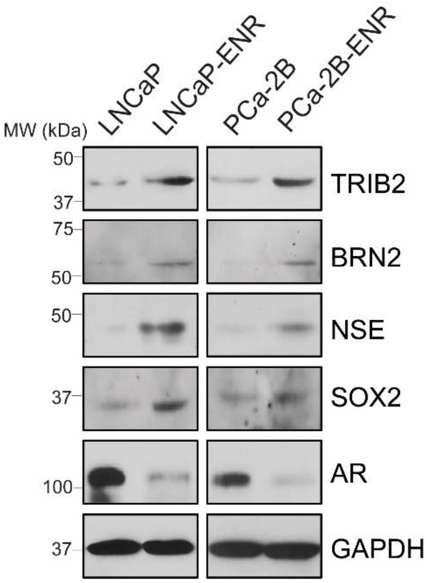

Fig. S8. Enzalutamide resistant prostate cancer cells express neuroendocrine markers. Western blot analysis showing protein levels of NE and stemness markers in the indicated cell lines. Positions of the molecular weight markers for the ladder are indicated along with the western blot data.

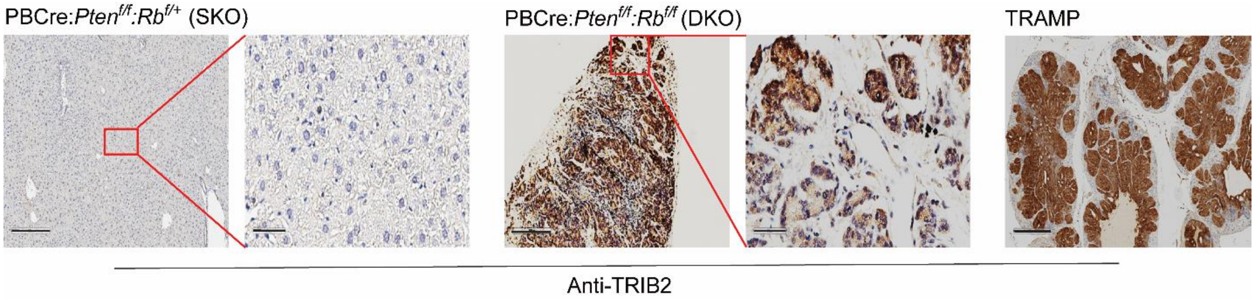

Fig. S9. Transgenic mouse models representing neuroendocrine prostate cancer phenotype expresses high levels of TRIB2. Paraffin-embedded tumor sections were stained with TRIB2 antibody as described before. SKO: Single knockout; DKO: Double knockout; Slides were kindly provided by Dr. David Goodrich at the Roswell Park Cancer Institute, Buffalo, NY **(Ref: 26).** The TRAMP (Transgenic Adenocarcinoma of the Mouse Prostate) tumors were dissected at the age of 28 weeks. These tumors show NE differentiation beyond 24 weeks of age. Slides were stained with rabbit monoclonal anti-TRIB2 antibody (Cell Signaling) showing strong expression of TRIB2. (Scale bar = 300µm; scale bar = 50µm (enlarged)).

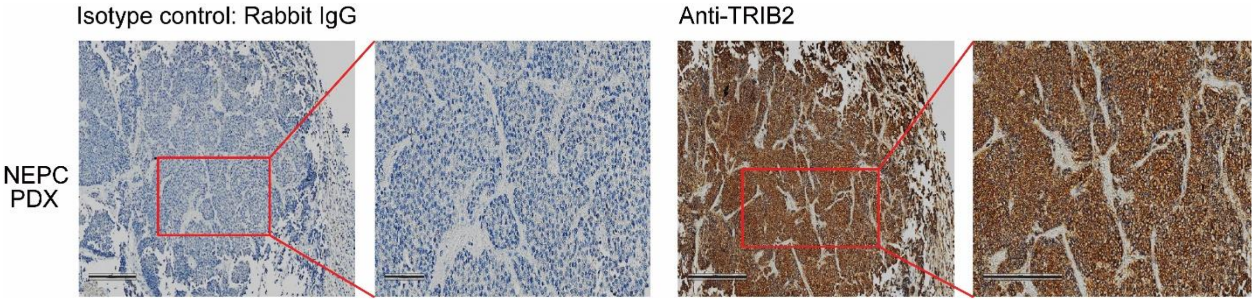

Fig. S10. Neuroendocrine prostate cancer patient-derived xenografts (NEPC-PDX) show high levels of TRIB2 proteins. Representative pictures from IHC analysis of NEPC-PDX tumors (Beltran #1078) stained with control IgG or anti-TRIB2 rabbit monoclonal antibody. Strong expression of TRIB2 was observed in all the NEPC tumor samples analyzed (n=3). (Scale bar = 300μm; scale bar = 200μm (enlarged)).

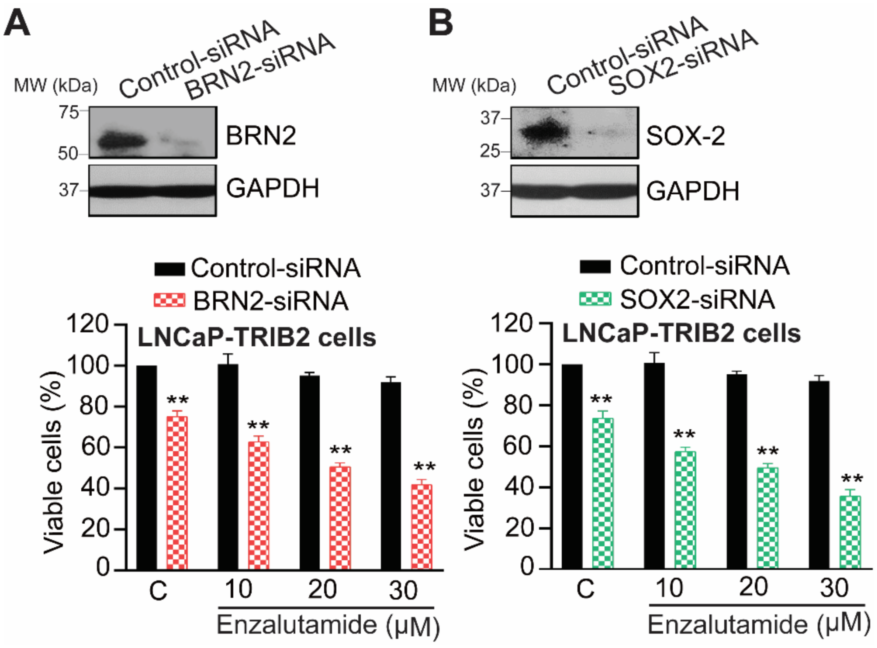

Fig. S11. Role of BRN2 and SOX2 in enzalutamide resistance. Whole cell lysates were analyzed by Western blot to evaluate the knockdown efficiency of **(A)** BRN2 siRNA or **(B)** SOX2 siRNA in LNCaP-TRIB2 cells. **(A, B)** Role of BRN2 and SOX2 in enzalutamide resistance was determined by treating TRIB2-OE cells with siRNAs and cell viability was measured by MTS/PES assay. Positions of the molecular weight markers for the ladder are indicated along with the western blot data. Two-way ANOVA, Tukey's multiple-comparison test was applied. Data presented as mean values ± SE. $^{**}p<0.005$.

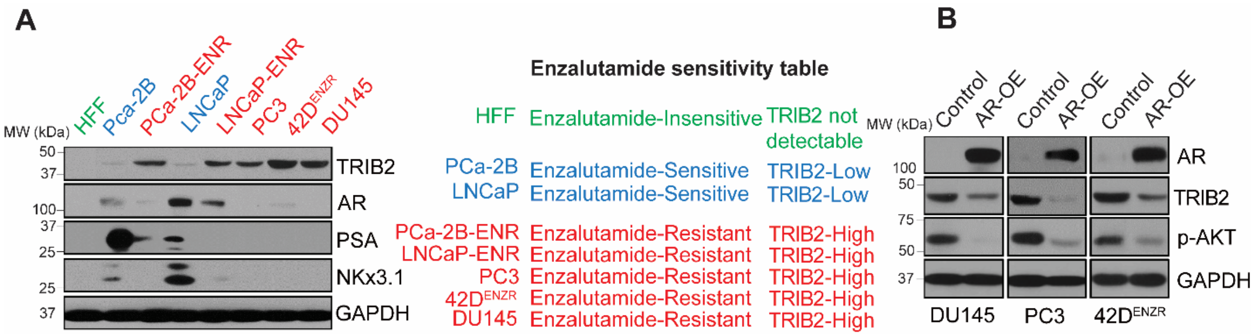

Fig. S12. TRIB2 is downregulated by AR activity. A) Whole cell lysates were analyzed by Western blot to detect levels of Trib2, AR and targets. Table showing relationship between Trib2 level and sensitivity to enzalutamide. High level of Trib2 in prostate cancer cells develop enzalutamide-resistance. **B)** AR null cells were transfected with full length human AR gene to overexpress (OE) AR proteins and treated with R1881 (10nM). Whole cell lysates were analyzed by Western blot to detect relative expression of AR and Trib2. Positions of the molecular weight markers for the ladder are indicated along with the western blot data.
